# Molecular mapping of a novel lesion mimic gene (*lm4*) associated with enhanced resistance to stripe rust in bread wheat

**DOI:** 10.1186/s12863-021-00963-6

**Published:** 2021-01-25

**Authors:** Rong Liu, Jing Lu, Shigang Zheng, Mei Du, Chihong Zhang, Minxiu Wang, Yunfang Li, Jiayi Xing, Yu Wu, Lei Zhang

**Affiliations:** 1 Chengdu Institute of Biology, Chinese Academy of Sciences, Chengdu, 610041 China; 2 University of Chinese Academy of Sciences, Beijing, 100049 China; 3 Innovative Academy for Seed Design, Chinese Academy of Sciences, Beijing, 100049 China

**Keywords:** Lesion mimic, Stripe rust resistance, Wheat, Programmed cell death

## Abstract

**Background:**

Lesion mimics (LMs) are disease-like symptoms that occur randomly on plant green leaves in the absence of pathogens. A previous study showed that LMs are related to enhanced resistance to a broad spectrum of diverse pathogen races and programmed cell death (PCD). Stripe rust is a globally epidemic fungal disease that can substantially reduce the quality and yield of crops. The development of resistant cultivars is an economical and environmentally friendly way to enhance the adaptability and yield stability of crops instead of the use of fungicide applications.

**Results:**

In this study, a novel LM gene affording *Pst* resistance was identified and mapped with molecular markers developed for marker-assisted selection (MAS)-based wheat breeding. In this study, a novel LM gene named *lm4*, which is closely linked (8.06 cM) to SSR markers *Xgwm210* and *Xgwm455*, was identified by using a Yanzhan 1/Neixiang 188 RIL population. The genetic distance of *lm4* was then narrowed such that it was flanked by SSR markers with 0.51 cM and 0.77 cM intervals. Two SSR markers, *lm4_01_cib* and *lm4_02_cib*, were developed based on the content in the Chinese Spring genome database and wheat 660 K SNP results; these markers can be used to conduct MAS of LMs in wheat. The results also showed that *lm4* significantly improved the resistance of stripe rust in wheat.

**Conclusions:**

Therefore, *lm4* is associated with stripe rust resistance, which may provide theoretical support for future crop disease-resistance breeding and for understanding the plant apoptosis mechanism.

**Supplementary Information:**

The online version contains supplementary material available at 10.1186/s12863-021-00963-6.

## Background

Lesion mimics (LMs), which are also referred to as hypersensitive reaction-like (HRL) traits, occur spontaneously in leaf tissue without attack by any plant pathogens. LMs may provide enhanced plant resistance to a broad spectrum of diverse pathogen races [1, 2]. LMs exhibit different phenotypes, such as their color and size, with respect to the timing and conditions [3]. Previous studies have reported that LM traits exist in several plant species, such as maize [4, 5], Arabidopsis [6, 7], barley [8], and rice [9]. Studies of lesion mimics have provided insight into the activation of programmed cell death (PCD) or defense response pathways in plants [10]. Some LM mutants spontaneously express defense response genes involved in plant disease resistance signaling pathways [3, 11].

LM mutants have also been reported to be resistant to virulent pathogen races, which supports the direct use of LM mutants in crop disease-resistance breeding [12]. However, only a few studies concerning lesion mimics in wheat have been reported [2, 10]. Previous studies have reported that the C591 mutant (M8) is a stable flecking mutant [13]. Another leaf flecking mutant (M66) showed enhanced resistance to powdery mildew, stripe rust and brown rust [14–16]. Kamlofski et al. reported a hypersensitive-like (HPL) trait that was similar to the lesion-mimic phenotype and enhanced resistance to leaf rust [12]. A dominant gene (*lm*) from wheat cultivar Ning 7840 was located on chromosome 1BL and provided resistance to leaf rust in adult plants [2]. Two light-dependent lesion-mimic genes (*lm1* and *lm2*) have been subsequently mapped onto 3BS and 4BL [17]. Wang et al. reported that a novel light-dependent lesion-mimic mutation (*lm3*) was closely linked to the SSR marker *Xbarc203* on chromosome 3BL, and the resulting wheat mutants exhibited enhanced resistance to powdery mildew [18]. To date, just a few lesion-mimic-related genes have been characterized in bread wheat. Our knowledge of the effects of LMs on wheat disease resistance is limited, and the chromosome locations of the genes underlying the LM trait have not been determined [2]. Therefore, characterizing LM genes and elucidating their functions is of great significance to understand both the whole signal transduction pathway of programmed cell death and disease resistance mechanisms in crop plants.

Stripe rust is an and airborne fungal disease caused by *Puccinia striiformis* f. sp. *tritici* (*Pst*) and occurs worldwide [19]. Stripe rust can significantly reduce the quality and yield of crops [20]. The development of cultivars exhibiting durable tolerance to various pathogens is an economical and environmental way to enhance the adaptability and yield stability of crops instead of the use of fungicide applications [21–24]. The objectives of this study were to (1) identify LM genes in wheat and map them, (2) investigate the probable effects of the lm gene on *Pst* resistance and important agronomic traits, and (3) develop molecular markers that are useful in MAS and gene cloning in the future.

## Results

### Phenotypic and genetic analysis of lesion mimics in the RIL population

**Fig. 1:**
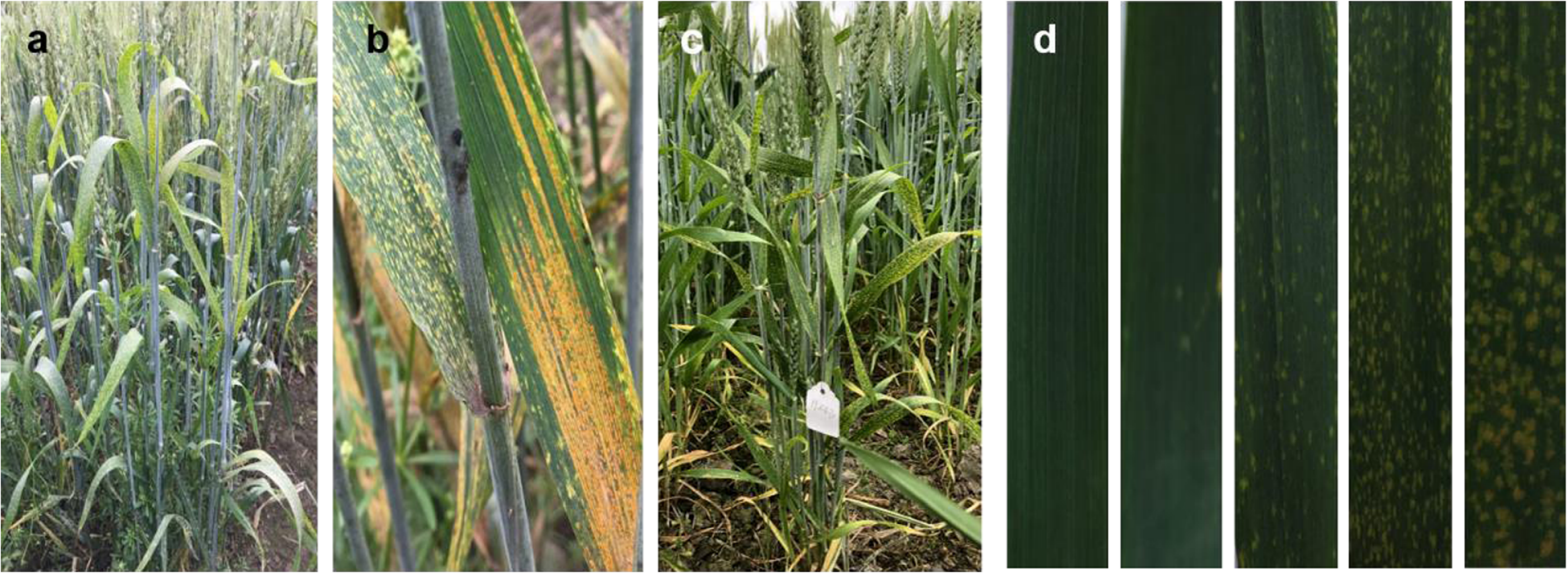
**a**, **b** Lesion mimic phenotype and stripe rust on wheat leaves; **c** The phenotype of F1 crossed by LM4 and LM0 (LM4 and LM0 wheat lines were from Yanzhan1/Neixiang188 RILs); **d** Phenotype and classification of LM

In this study, lesion-mimic (LM) traits likely appear as small yellow spots (disease-like symptoms) randomly spread throughout the green leaves of wheat (Fig. 1a, b, c). LMs appear without any plant pathogens, and LM spots started at approximately the fifth-leaf stage of wheat plants. In the current study, we found lesion-mimicking phenomena among Yanzhan 1/Neixiang 188 RILs. In the present study, the lesion-mimic trait was classified into scores of 0–4 based on the spread number and severity of yellow spots on the wheat leaves (Fig. 1d). According to their LM scores, all the RILs were then divided into two groups from 2015 to 2018: the normal-phenotype (LM0–1) group and the LM-phenotype (LM2–4) group. The segregation ratio of the two groups of LM traits in 2015–2018 was tested by the chi-square fitness test (Table 1). The segregation of the normal and LM phenotypes in the population conformed to a 1:1 ratio (*p* > 0.05) in all the environments. We crossed several LM4- and LM0-phenotype RILs, and the F1s showed lesion-mimic traits in their leaves (Fig. 1c). These results suggest that the lesion-mimic phenotype in Yanzhan 1/Neixiang 188 RILs is seemingly controlled by a single dominant gene. The dominant lesion-mimic gene identified in this study was named *lm4*.

**Table 1:** chi-square fitness test of the segregation ratio of LM phenotype

Year+ location	Normal phenotype	LM phenotype	Segregation ratio 1:1	
	LM0–1	LM2–4	*P* value	χ^2^
2015Shifang	103	95	0.57	0.32
2016Shifang-1	109	89	0.16	2.02
2016Shifang-2	117	81	0.01	6.55
2017Shifang	97	101	0.78	0.08
2017Maerkang	91	107	0.26	1.29
2018Shifang	94	102	0.57	0.33

2016Shifang-1 and 2016Shifang-2 were two replicates planted in different fields in Shifang

### Relation between lesion mimics and stripe rust resistance

In this study, a significant negative correlation was investigated between the lesion-mimic score and stripe rust IT value in 2016–2017 (r = − 0.53 ~ − 0.66, *p* < 0.01) (Table 2). As the degree of LM increased, the wheat stripe rust IT value significantly decreased. In breeding programs, plants with LMs are essentially highly resistant to stripe rust. Therefore, these results indirectly indicate that *lm4* plays an important role in wheat stripe rust resistance, which can provide new insights or theoretical support for future disease-resistance breeding.

**Table 2:** Correlation between the phenotype of LM and IT value of stripe rust in 2016–2018

Year- location	Correlation coefficient	Normal phenotype		LM phenotype		
		LM0	LM1	LM2	LM3	LM4
2016Shifang	−0.53	3.2 ± 1.0^a^	3.6 ± 0.7^a^	3.1 ± 0.9^a^	2.1 ± 0.8^b^	1.6 ± 0.5^b^
2016Shifang	−0.67	3.3 ± 1.0^a^	3.3 ± 0.8^a^	2.3 ± 1.1^b^	2.1 ± 0.6^bc^	1.5 ± 0.5^c^
2017Shifang	−0.61	3.0 ± 1.4^a^	3.1 ± 1.0^a^	2.8 ± 1.5^a^	1.0 ± 0.9^b^	0.9 ± 0.8^b^
2017Maerkang	−0.66	3.3 ± 1.3^a^	3.2 ± 1.4^a^	2.6 ± 1.7^a^	0.5 ± 0.3^b^	0.2 ± 0.1^b^

Different letters represent the significance difference, *p* < 0.05

### Lesion-mimic effects on agronomic traits

The effects of lesion mimics on the agronomic traits of wheat plants were investigated at Shifang and Maerkang in 2016–2018 in Sichuan Province (Table 3). Except for plant height (PH) in 2018 there were no significant effects of lesion mimics on spikelet number (SPI), number of sterile spikelets per spike (SSNS), grain number per spike (GNS), 1000-grain weight (TGW), or spike length (SL) of wheat. In general, these results reflect that LMs have no impact on agronomic traits of wheat, including yield traits.

**Table 3:** 2016–2018 comparison of agronomic traits at different LM levels in the YZ1/NX188 RILs

Traits/Years	Normal phenotype		LM phenotype		
	LM0	LM1	LM2	LM3	LM4
PH (cm)					
2016	77.9 ± 14.9^a^	72.3 ± 15.3^a^	76.6 ± 15.6^a^	75.7 ± 10.6^a^	76.8 ± 14.2^a^
2017	71.7 ± 12.0^a^	76.5 ± 12.0^a^	74.4 ± 12.9^a^	70.7 ± 9.8^a^	72.7 ± 12.6^a^
2018	72.1 ± 12.5^a^	78.9 ± 11.3^ab^	85.9 ± 19.3^b^	75.4 ± 14.8^ab^	70.8 ± 12.5^a^
SL (cm)					
2017	9.0 ± 2.4^a^	9.4 ± 1.3^a^	9.1 ± 1.4^a^	9.0 ± 1.6^a^	8.9 ± 1.8^a^
2018	9.1 ± 2.1^a^	9.0 ± 1.2^a^	11.1 ± 6.9^a^	9.3 ± 1.1^a^	9.5 ± 2.7^a^
SPI					
2016	21.0 ± 2.2^a^	20.9 ± 2.1^a^	20.8 ± 1.7^a^	21.0 ± 1.9^a^	21.2 ± 1.7^a^
2017	16.7 ± 4.2^a^	17.7 ± 3.4^a^	17.2 ± 3.3^a^	17.2 ± 3.5^a^	16.8 ± 3.7^a^
2018	21.1 ± 2.3^a^	20.8 ± 2.5^a^	21.3 ± 2.2^a^	21.7 ± 3.0^a^	21.3 ± 2.2^a^
GNS					
2016	43.6 ± 6.2^a^	42.8 ± 2.0^a^	41.9 ± 5.3^a^	42.4 ± 5.0^a^	42.4 ± 7.2^a^
2017	40.0 ± 7.1^a^	42.4 ± 9.2^a^	41.5 ± 7.8^a^	42.1 ± 6.8^a^	40.6 ± 8.6^a^
2018	46.4 ± 7.4^a^	46.7 ± 9.2^a^	45.6 ± 15.1^a^	46.0 ± 10.0^a^	45.7 ± 6.4^a^
SSNS					
2016	1.9 ± 1.1^a^	1.9 ± 0.8^a^	2.2 ± 0.9^a^	1.7 ± 0.8^a^	2.3 ± 1.6^a^
2017	1.3 ± 0.6^a^	1.3 ± 0.7^a^	1.2 ± 0.8^a^	1.2 ± 0.6^a^	1.3 ± 0.6^a^
2018	1.8 ± 0.7^a^	1.8 ± 0.8^a^	1.7 ± 0.9^a^	1.8 ± 0.6^a^	2.0 ± 0.6^a^
TGW(g)					
2016	36.9 ± 6.8^a^	36.3 ± 7.9^a^	37.0 ± 6.8^a^	39.2 ± 6.3^a^	36.3 ± 7.4^a^
2018	49.1 ± 4.9^a^	50.4 ± 3.8^a^	47.8 ± 0.7^a^	49.8 ± 0.3^a^	45.2 ± 5.5^a^

^a^ Different letter represent the significance difference, *p* < 0.01, plant height (PH), spikelet number (SPI), number of sterile spikelets per spike (SSNS), grain number per spike (GNS), 1000-grain weight (TGW), spike length (SL)

### Chromosomal location of the lesion-mimic gene

**Fig. 2:**
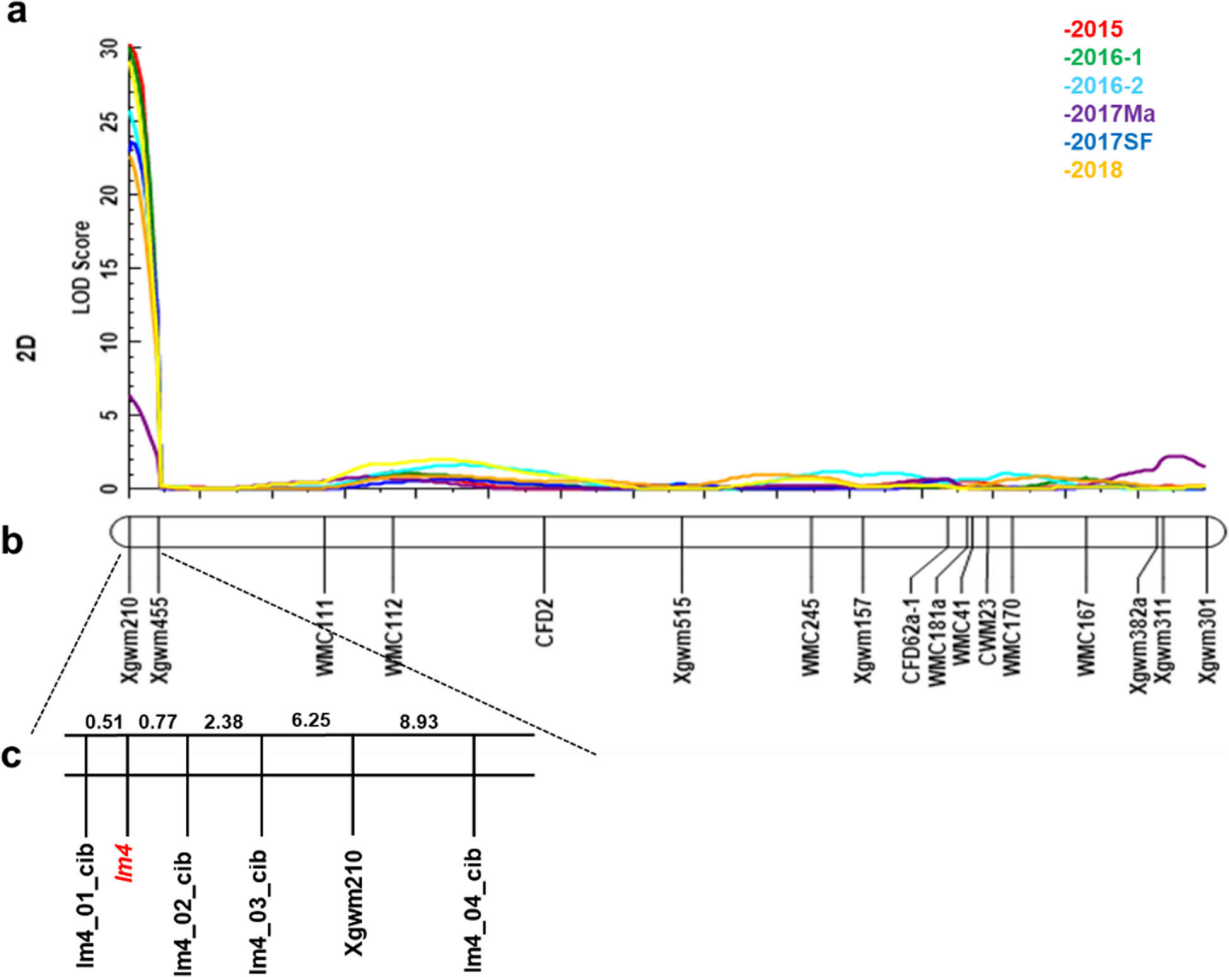
The genetic map of the region around lm4 on chromosome 2DS. LOD curves with data from different years (2015–2018 Shifang and Maerkang) (**a**), initial (**b**) and advanced (**c**) genetic linkage maps

A total of 252 SSR markers were used to construct a genetic linkage map of 198 RILs (linkage map obtained from CAAS) in this study. *lm4* was preliminarily localized to 2DS. However, the genetic distance was not close (8.06 cM), and the gene was flanked by SSR markers *Xgwm210* and *Xgwm455* (Fig. 2a, b); the LOD value was 30.1, and the phenotypic variation explained (PVE) was 50.8% (Fig. 2a). Therefore, a wheat 660 K SNP array was used to develop new molecular markers to narrow the physical genetic distance. Several SSR primers (markers) linked to *lm4* on the 2DS chromosome were developed from the results of the wheat 660 K SNP array (Table S1, S2, Additional file 1). The genetic distance of *lm4* was then narrowed such that the gene was flanked by SSR markers *lm4_01_cib* and *lm4_02_cib* (Fig. 2c); the genetic distances were 0.51 cM and 0.77 cM, respectively. The LOD value was 19.4, and the PVE was 37.1%. *lm4* was ultimately delimited to an approximately 50 Mb regions on the basis of the Chinese Spring chromosome 2D genome sequence. The *lm4* gene identified in this study is a novel lesion-mimic gene, which is different from the previously reported LM gene.

## Discussion

### A novel lesion-mimic gene and mapping

Lesion mimics constitute a disease-like phenomenon that occurs in plant leaves without any pathogen infection, injury or obvious stress [25]. The phenotype of LMs in the current study is similar to that of yellow spot lesions on wheat leaves and lesion spots at booting in the fifth or sixth leaf stage of wheat. The previously reported LM gene in wheat, *lm*, was located on 1BL [2], *lm1* and *lm2* were located on 3BS and 4AL [17], and *lm3* was mapped onto 3BL [18]. Unlike the previously reported lesion-mimic genes, the LM gene found in this study (*lm4*) is a novel type of lesion-mimic gene in wheat; this gene was mapped to 2DS and is a dominant LM gene derived from Yanzhan 1/Neixiang 188 RILs. The phenotype of the lesion mimic (*lm4*) in this study was also different from that of the HLP mutant induced by EMS [12]. Although LM traits are expressed at about the fifth or sixth leaf stage of wheat, LMs are the result of a natural mutation, and the types of lesion manifested also differed in this study: the LM phenotype induced by EMS involves small white spots (1 ~ 2 mm) on the leaves [12]. Therefore, LMs constitute a novel type of lesion-mimic trait that is different from that of the EMS-induced mutant, and *lm4* is also different from previously reported LM genes.

### Relationships between LMs and yield traits in wheat

Previous studies have shown that most lesion mimics have a negative effect on agronomic crop traits, especially those affecting yield production, although HLP mutants have been excluded [12, 16, 26]. In the current study, the agronomic traits, including yield traits (SPI, SSNS, GNS and TGW), of the wheat RILs were not significantly reduced by the appearance of a lesion-mimic phenotype compared with the phenotype of normal wheat lines (Table 3). Breeders aim to develop disease-resistant and high-yielding crop varieties. In this study, we found that *lm4* significantly improved stripe rust resistance in wheat and did not affect major yield-related traits. Thus, this gene could be used as a potential tool for future disease-resistance breeding.

### Effects of lesion mimics on stripe rust resistance

Stripe rust is a major fungal disease that threatens the quality and yield of wheat [27]. Controlling the spread of stripe rust and breeding new resistant varieties to improve the quality and yield of wheat is the main goal of breeders. In addition to disease resistance genes for specific races, studies on certain disease resistance-related genes have gradually attracted increased amounts of attention in recent years. Li et al. reported that *lm* (derived from Ning 7840 and located on 1BL) can enhance leaf rust resistance in wheat [2]. *lm1* and *lm2* were mapped to 3BS and 4AL, respectively, and are correlated with improved powdery mildew resistance [17]. The recently located LM gene *lm3* (mapped onto 3BL) provides resistance to powdery mildew in adult plants [18]. These results provide new insight into the molecular mechanism of LM to improve broad-spectrum resistance in wheat, which may be helpful for screening candidate genes underlying the LM trait in this species. In this study, *lm4* was found to be a novel lesion-mimic gene that is related to enhancing stripe rust resistance in wheat. Certain QTLs for stripe rust, Fusarium head blight resistance and leaf rust have been reported to be located on 2DS in wheat, close to *lm4* [28–33]. In this study, a significant correlation was found between LMs and stripe rust resistance in the field (r = − 0.61, *p* < 0.01). The potential functions of *lm4* in response to the above diseases in wheat deserve to be further studied.

### Signaling pathways related to lesion mimics

In recent years, studies have reported that lesion mimics resembling a hypersensitivity reaction may enhance the resistance of plants by certain defense signaling pathways involved in plant disease resistance or stress resistance. Some LMs are associated with the production of ROS, which respond to cell death signals [3, 34, 35]. The inducible defense response of plants exists mainly to provide plants with an optimal defense system by relying on signaling pathways, such as those involving salicylic acid, jasmonic acid and ethylene, and the cross-talk between them. The salicylic acid-dependent pathway leads to cell death. In plants, cell death may play an important role in resistance to pathogens [36].

In addition, studies have reported that LMs may be associated with the programmed cell death signaling pathway. Pathogens have difficulty invading necrotic spots; therefore, LMs could improve plant disease resistance [37]. Plants have complex systems for regulating cell death, and these systems have a purpose in plant development against pathogens and environmental stress [37]. These results indicate that the mechanism of LM-enhanced plant resistance may be caused by associations with resistance genes or may involve signaling pathways to regulate plant defense responses and the programmed cell death pathway in plants. In this study, we mapped *lm4* to a 50 Mb interval on 2DS and identified 18 predicted candidate genes (Table S3). Among these candidate genes, *TraesCS2D02G090600* is related to the physiological defense response and immunity-related protein activity; *TraesCS2D02G091200* and *TraesCS2D02G092200* are involved in regulating cell death and defense upon pathogen recognition; and *TraesCS2D02G090900* and *TraesCS2D02G091100* are related to signal transduction. *TraesCS2D02G091000*, *TraesCS2D02G091600*, *TraesCS2D02G091900* and *TraesCS2D02G092100* function in response to stimuli, and *TraesCS2D02G091300* and *TraesCS2D02G091400* are related to leaf senescence and chloroplasts, respectively.

Lesion-mimic mutants can be a powerful tool to study their involvement in cell death. In addition to this genetic approach, physiological and biochemical characterization of the corresponding proteins was performed to identify the function of LM genes. This work should provide insight into cell death, defense or development through the determination of the biochemical functions of these proteins, their subcellular localization and their interacting proteins [3]. In the present study, fine mapping or gene cloning are needed for an improved understanding of the resistance mechanism and function of *lm4*. Studying the LM gene and its function is crucial for understanding the signaling pathways involved in plant apoptosis and disease resistance mechanisms.

## Conclusions

A novel lesion-mimic gene (*lm4*) was identified by using a Yanzhan 1/Neixiang 188 RIL population. This gene is closely linked to SSR markers *lm4_01_cib* and *lm4_02_cib*, separated by 0.51 cM and 0.77 cM, respectively, intervals on 2DS. SSR markers were developed based on the content of Chinese Spring genome database and wheat 660 K SNP results, and these markers can be used for MAS of LM in wheat. In this study, we found that LMs were related to enhanced resistance to stripe rust in wheat. Therefore, resistance-related gene mapping (cloning) or resistant-cultivar breeding is an economical and environmentally friendly way to enhance the adaptability and yield stability of crops instead of the use of fungicide applications. In the present study, *lm4* was associated with stripe rust resistance, and 18 candidate genes were chosen to analyze potential functions. LM gene cloning is required to understand the functions and disease resistance mechanism in wheat. This study may provide new ideas or theoretical support for future crop plant disease-resistance breeding and for understanding the plant apoptosis mechanism.

## Methods

### Plant materials

A total of 198 wheat recombinant inbred lines (RILs) of the Yanzhan 1 × Neixiang 188 mapping population (obtained from the Chinese Academy of Agricultural Sciences [CAAS]) were used for linkage analysis. The mapping population for this study was planted at the experimental station of the Chengdu Institute of Biology, Chinese Academy of Sciences, in Shifang (SF) and Maerkang (Ma) during the growing seasons of 2015 to 2018, according to local legislation in Sichuan Province (2015–2016, 2016–2017, 2017–2018 at SF; 2016–2017 and 2017–2018 at Ma). Twenty seeds of each accession were planted in a row. The stripe rust-susceptible wheat line Minxian 169 (obtained from the Chengdu Institute of Biology, Chinese Academy of Sciences), a control, was inserted after every 9 rows. Each experiment was arranged in a randomized complete block design, with two replicates, at Shifang and Maerkang from 2015 to 2018.

### Evaluations of lesion-mimic phenotypes and agronomic traits

In total, 198 RILs and 2 parents (Yanzhan 1 and Neixiang 188) were evaluated for their lesion-mimic (LM) phenotype at SF (104°17′ E, 31°13′ N) and Ma (102°11′ E, 31°92′ N) in Sichuan Province from 2015 to 2018. The lesion-mimic phenotypes were arbitrarily subdivided into 5 scores based on flag leaf symptoms according to the methods of Yao et al. [17], with modifications. No visible lesions (specks) were recorded as 0 (the parental phenotype); few specks and low severity (< 25%) were recorded as 1; some specks and moderate severity (25–50%) were recorded as 2; large specks and high severity (50–75%) were recorded as 3; and a large number of specks and very high severity (> 75%) were recorded as 4. Plants with scores of 0 or 1 were considered normal, and those with scores of 2 or higher were classified as having lesion-mimic phenotypes.

Agronomic traits of the RILs were investigated by our team at the Chengdu Institute of Biology, Chinese Academy of Sciences, during the Shifang cropping seasons. These parameters included plant height (PH), spikelet number (SPI), number of sterile spikelets per spike (SSNS), grain number per spike (GNS), 1000-grain weight (TGW), and spike length (SL). Three to five plants of each wheat line were evaluated, and their means were used for analysis.

### Evaluation of stripe rust resistance

All 198 RIL lines and the 2 parents were evaluated for stripe rust at Shifang and Maerkang from 2016 to 2018. Mixtures of *Pst* spores from races *Pst-CYR32*, *Pst-CYR33*, *Pst-SU11*, *Pst-Hybrid46* and *Pst-G22* (provided by SAAS) were suspended in 0.05% Tween 20 and sprayed onto four-leaf-stage wheat seedlings.

In the adult stage, stripe rust response types (ITs) were identified, and each environment was evaluated at least twice, mainly from 20 weeks to 23 weeks after sowing. Stripe rust infection types (ITs) were evaluated based on typical 0–4 classification systems [38].

### Lesion-mimic gene mapping

Seedling leaves of the 198 RIL lines and two parents (Yanzhan 1, Neixiang 188) were collected, and genomic DNA was extracted from each sample using the CTAB method [39]. The quality and quantity of the DNA were determined using 1.0% agarose gel electrophoresis and a spectrophotometer (NanoDrop ND-1000, Thermo Scientific, Wilmington, DE). Two hundred and fifty-two polymorphic SSR markers covering 21 wheat chromosomes were used to genotype the mapping population to identify the chromosomal location of the LM gene (Additional file 1). Information about the SSR markers is available on the Grain Genes website (http://wheat.pw.usda.gov).

Based on the phenotypic evaluations, 10 wheat lines with an LM score of 0 and 10 RILs with an LM score of 4 were used to prepare two bulks representing extreme phenotypes. The DNA of these lines along with the parental lines was genotyped by 660 K SNP arrays at China Golden Marker Corporation (Beijing; http://www.cgmb.com.cn). Various SNP markers located on 2DS associated with lesion mimics were identified from the SNP typing results (Additional file 1). Whole wheat genome sequences were searched by SNP-tagged probe sequences (https://www.ncbi.nlm.nih.gov/) or according to the possible physical intervals on 2DS obtained from the SNP analysis search of the Chinese Spring genomic intervals (https://urgi.versailles.inra.fr/jbrowseiwgsc/gmod_jbrowse/?data=myData%2FIWGSC_RefSeq_v1.0&loc=chr2D%3A1..651852609&tracks=DNA&highlight=). A matched scaffold sequence was obtained, and repeated a DNA analysis was performed using the SSR Hunter 1.3 program (Li Qiang and Wan Jianmin 2005). The DNA sequences of both ends of the repeats were obtained, and primers were designed using Primer Premier 6.0 software (Canada). These primers (Table S1) were used for PCR- and electrophoresis-based analyses, and primers suitable for polymorphism were selected as molecular markers to obtain genotypes in the genetic population (Table S2).

PCR was conducted in a total volume of 20 μl comprising 200 ng of DNA template, 10 μl of 2× Es Taq MasterMix (Kangwei Century, China), 0.6 μl of 10 μM forward primer and 0.6 μl of 10 μM reverse primer. The amplification procedure was as follows: 94 °C for 5 min; 35 cycles of denaturation at 94 °C for 30 s, 45–60 °C (adjusted according to the primers) for 30 s, and 72 °C for 45 s; and then 72 °C for a total extension of 10 min. The separation of the PCR products was carried out by 1% agarose gel electrophoresis or 8% nondenaturing polyacrylamide gel electrophoresis. The agarose gel electrophoresis was performed with ethidium bromide (EB), and the polyacrylamide gel electrophoresis was performed with silver nitrate [40, 41].

### Data analysis

All phenotypic data were recorded in Microsoft Office Excel 2013 for statistical analysis. One-way analysis of variance (ANOVA) was conducted to evaluate the variance and significance between groups by using SPSS 20.0 and GraphPad Prism 5.0. The genetic segregation ratio of normal (LM0–1) and lesion-mimic phenotypes (LM2–4) was tested by the chi-square test. Mean phenotypes of LMs and stripe rust scores for each RIL collected from each individual experiment were used for QTL analysis. The inclusive composite interval mapping of additive (ICIM-ADD) QTL method was used, and a walking speed of 1.0 cM with a stepwise regression probability of 0.001 was chosen for QTL detection. The threshold for declaring a significant QTL was determined by 1000-permutation tests. The LOD score to determine significant QTLs was 3.5 in all environments, and a LOD threshold of 3.5 was the criterion selected for a significant QTL. Linkage map construction and QTL mapping were performed using QTL IciMapping V4.1 software, and the genetic distance between markers was measured using centimorgans (cM) [42]. The threshold of the logarithm of odds value was set to 3.0 to determine linkage between markers, with a maximum recombination fraction at 0.4.

## Supplementary Information

**Additional file 1.**

**Additional file 2.**

## Data Availability

The dataset and materials presented in the investigation are available from the supplementary tables and additional file 1.

## Abbreviations

LM: Lesion mimic
PCD: Programmed cell death
MAS: Marker-assisted selection
HRL: Hypersensitive reaction-like
RIL: Recombinant inbred line
PVE: Phenotypic variation explained
PH: Plant height
SPI: Spikelet number
SSNS: Number of sterile spikelets per spike
GNS: Grain number per spike
TGW: 1000-grain weight
SL: Spike length
ITs: Infection type
Yr: Stripe rust or yellow rust
Pst: *Puccinia striiformis* f. sp. *tritici*
